# Episcleritis in a patient with mucosal melanoma treated with interferon alfa-2b and radiotherapy: a case report

**DOI:** 10.1186/s13256-018-1913-7

**Published:** 2018-12-23

**Authors:** Li Yang, Shaofei Ji, Liping Wang, Yi Zhang

**Affiliations:** 1 Biotherapy Center, The First Affiliated Hospital of Zhengzhou University, No. 1 Jianshe East Road, Zhengzhou, 450052 China; 2 Department of Radiology, Orthopedics Hospital of Zhengzhou City, Zhengzhou, 450052 China; 3 Department of Oncology, The First Affiliated Hospital of Zhengzhou University, Zhengzhou, 450052 China

**Keywords:** Mucosal melanoma of the head and neck (MMHN), Episcleritis, Interferon alfa-2b, Radiotherapy, Immune-mediated, Anti-tumor immunity, Tumor immunology

## Abstract

**Background:**

Mucosal melanoma of the head and neck is a rare malignant tumor associated with a poor prognosis. Surgery, chemotherapy, radiotherapy, and biotherapy are common strategies for treating mucosal melanoma of the head and neck. Episcleritis is an idiopathic, immune-mediated disease, and is classified into two types: simple episcleritis and nodular episcleritis.

**Case presentation:**

In this case report we describe ocular changes involving simple episcleritis in a 65-year-old Chinese man with mucosal melanoma of the head and neck after treatment with interferon alfa-2b and radiotherapy. On the third day of interferon alfa-2b treatment, he began to develop simple episcleritis in his left eye. Moreover, the percentage of CD3^+^ T cells in lymphocytes from blood was increased after interferon alfa-2b treatment. After approximately 6 days, the symptoms of eye pain, hyperemia, and edema disappeared gradually. Then, after radiotherapy was performed three times, he again developed episcleritis in his left eye. The same symptoms of hyperemia and edema occurred again; CD3^+^ T cell frequency was also at a higher level. After approximately a week, all the symptoms disappeared completely. Simple treatment involving topical ofloxacin and phenylephrine was administered during the two periods of episcleritis.

**Conclusion:**

Episcleritis in this patient might have been due to the treatment with interferon alfa-2b and radiotherapy, leading to an increase in the level of CD3^+^ T cells and activation of immune system cells, which provides the guide for clinical clinicians.

## Background

Mucosal melanoma of the head and neck (MMHN) is a rare malignant tumor with a poor prognosis [1, 2]. Resection with a clear margin is the most important factor for good local control and better prognosis [3]. However, radical surgery is not possible in certain cases because the tumor may be located near important structures. Effective chemotherapy has not yet been established, and a common therapeutic strategy has not been clarified excluding curative surgical resection.

Currently, interferon alfa-2b (IFN-α2b) and radiotherapy are used to treat patients with MMHN in clinic. Many studies have reported that IFN-α2b and radiotherapy have little effect on the melanoma cells themselves, but instead activate immune-system cells to fight the disease [4, 5]. Episcleritis can occur as an isolated condition confined to the eye or it can also be associated with an idiopathic, immune-mediated disease [6]. We report the case of a patient with MMHN who developed episcleritis after adjuvant treatment with IFN-α2b and radiotherapy.

## Case presentation

A 65-year-old Chinese man was hospitalized in November 2012 for epistaxis since the previous month. Indirect nasopharyngoscopy revealed a scarlet mass with a rough surface in his left nasopharyngeal cavity. A clinical examination revealed no lymph node enlargement. He underwent surgery. A pathological examination indicated a mucosal melanoma in his left nasopharyngeal cavity. The results of computed tomography (CT) and MRI examinations showed that there was no metastasis to other organs.

This patient was hospitalized again in March 2013 to undergo adjuvant treatment, including IFN-α2b treatment, radiotherapy, and chemotherapy. A high dose of IFN-α2b (20 MIU/m^2^ per day) was administered for 5 days, then radiotherapy was performed 14 times (total 28 Gy), followed by a low dose of temozolomide (75 mg/m^2^ per day) for 3 weeks.

**Fig. 1:**
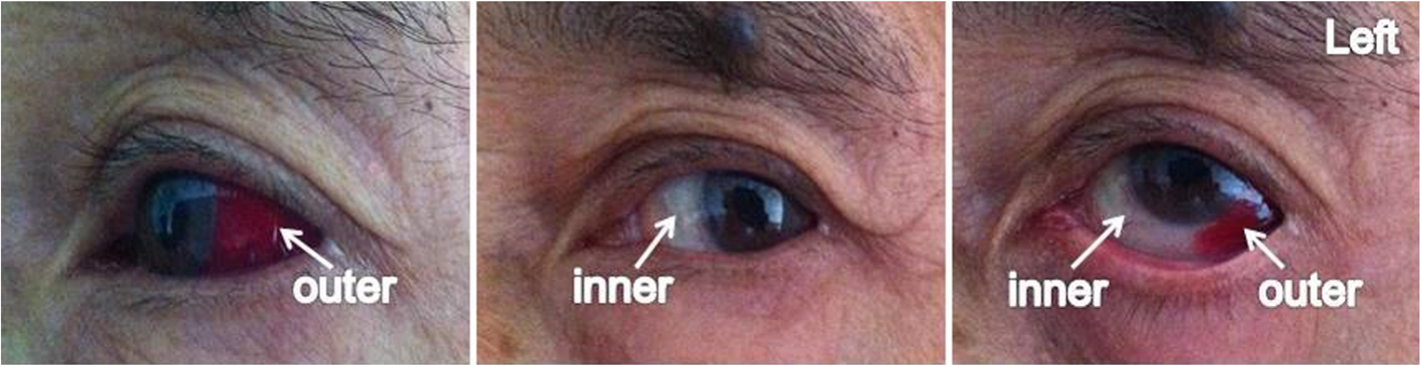
Photos of simple episcleritis in a patient after treatment with IFN-α2b. Simple episcleritis was visible on the lateral sclera and conjunctiva in the left eye of a patient with MMHN after treatment with IFN-α2b. The symptoms of severe hyperemia and edema were present in the outer quadrant of the left eye

**Fig. 2:**
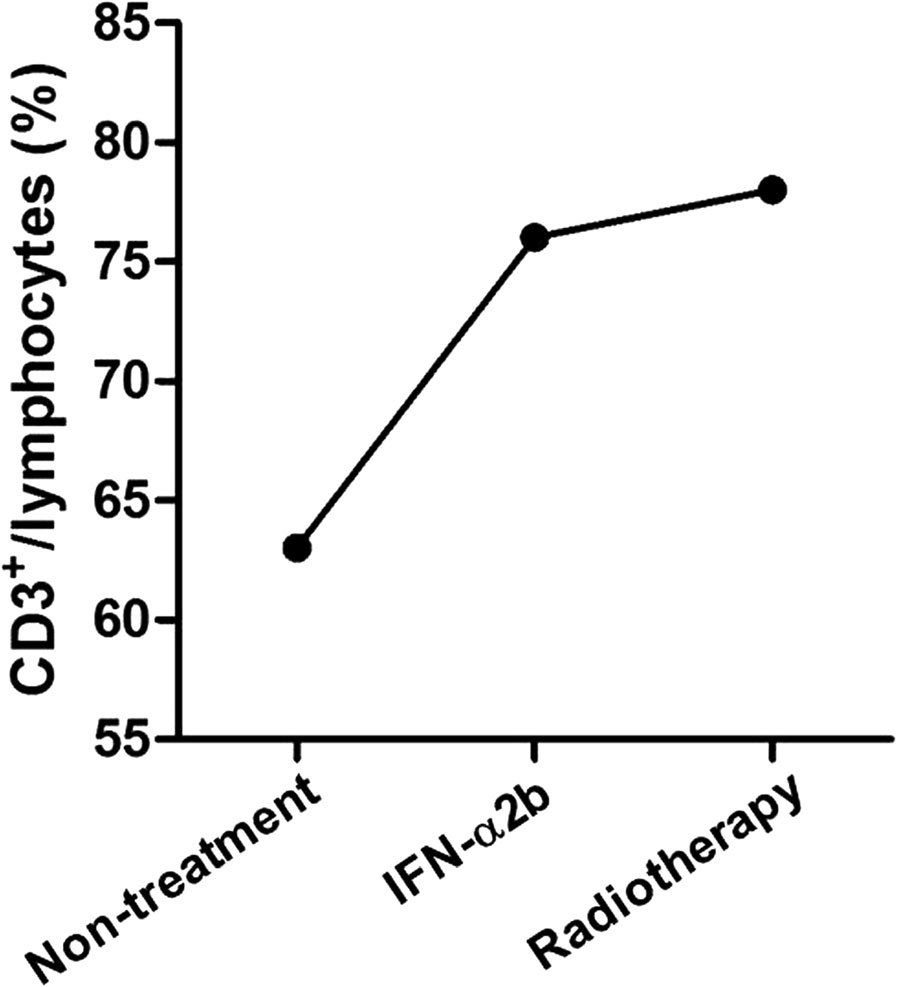
The percentage of CD3^+^ T cells before and after treatment with IFN-α2b and radiotherapy. CD3^+^ T cell frequency in lymphocytes from blood of this patient was analyzed by flow cytometry before and after treatment with IFN-α2b and radiotherapy. *IFN-α2b* interferon alfa-2b

**Fig. 3:**
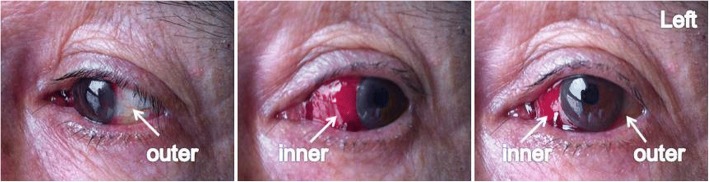
Photos of simple episcleritis in a patient after treatment with radiotherapy. Simple episcleritis was visible again in the left eye from this patient with MMHN after treatment with radiotherapy. The symptoms were similar to which were treated with IFN-α2b, but were limited to the inner quadrant

On the third day of IFN-α2b treatment, our patient began to develop simple episcleritis in his left eye. He experienced symptoms of severe eye pain, hyperemia, and edema on the lateral sclera and conjunctiva of his left eye. The redness was diffuse, and it covered a pie-shaped area confined to the outer quadrant (Fig. 1). We performed the phenylephrine blanching test to diagnose episcleritis. Episcleritis may be differentiated from scleritis by using phenylephrine eye drops, which cause blanching of the blood vessels in episcleritis, but not in scleritis. The percentage of CD3^+^ T cells in lymphocytes from blood was increased after IFN-α2b treatment (Fig. 2). After approximately 6 days, the symptoms of eye pain, hyperemia, and edema disappeared gradually. The treatment of IFN-α2b had been stopped when the symptoms disappeared. After radiotherapy was performed three times, our patient again developed episcleritis in his left eye, but it was limited to the inner quadrant. The same symptoms of hyperemia and edema occurred again (Fig. 3); CD3^+^ T cell frequency was also at a higher level (Fig. 2). After approximately a week, all the symptoms disappeared completely, and the treatment with radiology was still on-going. Simple treatment involving topical ofloxacin and phenylephrine was administered during the two periods of episcleritis. Whether the occurrence of these two episodes of episcleritis is associated with treatment of IFN-α2b and radiotherapy is of interest and worth studying.

This patient was followed up to detect late metastasis or recurrence for the first 2 years after surgery and the adjuvant therapy, and no metastasis or recurrence was found.

## Discussion

Melanoma has become one of the most prevalent cancers in fair-skinned populations and is now regarded as the fifth most common cancer in men and the sixth most common cancer in woman in the USA [7]. The highest recorded incidence of melanoma worldwide is in Queensland (Australia) with an annual incidence of 55.8 cases per 100,000 men and 41.1 cases per 100,000 women. Along with the increased incidence rate, there has been an increase in melanoma-related mortality.

Men are approximately 1.5 times more likely to develop melanoma than women are [8]. The most common sites of occurrence of cutaneous melanoma are sex-dependent: the back for men and the arms and legs for women. The most important and potentially modifiable environmental risk factor for developing malignant melanoma is exposure to ultraviolet rays because of their genotoxic effect [9]. The most important host risk factors are the number of melanocytic nevi, familial history, and genetic susceptibility [8]. Clinical staging is based on the history and a physical examination that includes the locoregional area and draining lymph nodes, complete skin examination, histopathologic microstaging, imaging, and sentinel lymph node biopsy [9].

MMHN is a rare malignant tumor with a poor prognosis [1]. It usually arises in the nasal cavity, paranasal sinuses, and oral cavity [10]. The most common cause of death is distant metastasis. T stage, neck lymph node status, and surgical margins are independent prognostic factors for the overall survival in patients with MMHN [11]. Multimodality treatment may improve survival [12]. Surgery, chemotherapy, and radiotherapy are common strategies for treating MMHN, but the control of metastasis is difficult. An accepted therapeutic strategy has not yet been developed for treating patients with MMHN after excluding the possibility of curative surgical resection. The use of immunotherapy remains uncommon for MMHN. Bartell *et al.* [13] reported that biochemotherapy for advanced MMHN should be considered a systemic treatment option for patients with this aggressive malignancy.

Episcleritis is a benign recurring condition; it is a self-limiting inflammatory disease affecting part of the eye called the episclera [14]. Episcleritis is classified into two types: simple episcleritis and nodular episcleritis [14]. Simple episcleritis lesions are flat. Nodular episcleritis lesions have raised surface. Jabs *et al.* [15] suggested that episcleritis was most often a mild ocular disorder, unassociated with ocular complications, and that it responded to topical medications.

No clear conclusions have been drawn as to the etiology of episcleritis [15]. Episcleritis can occur as an isolated condition confined to the eye or it can also be associated with an idiopathic, immune-mediated disease [6]. The diagnosis of episcleritis is based on the history and a physical examination. Episcleritis may be differentiated from scleritis by using phenylephrine or Neo-Synephrine® (phenylephrine) eye drops, which causes blanching of the blood vessels in episcleritis, but not in scleritis [14]. Treatment is unnecessary in episcleritis, because episcleritis is a self-limiting condition.

IFNs are low-molecular-weight glycoproteins that possess antiviral, antiproliferative, pro-apoptotic or anti-apoptotic, and immunomodulatory properties [16]. We distinguish between type I IFNs, which include several proteins called IFN-α and a single protein, IFN-β (which binds to the same specific receptor), and type II IFN-γ (which binds to multiple receptors). Type I IFNs stimulate the activity of natural killer and T cytotoxic cells, and increase the expression of the major histocompatibility complex class I. In addition, they stimulate the development of type 1 T helper cells, especially in humans [16]. IFN-α is mainly produced by plasmacytoid dendritic cells in response to viral infection [17].

Recent studies have shown that IFN-α, an important drug used in the treatment of malignant melanoma, has little effect on the melanoma cells themselves, but instead activates immune system cells to fight the disease [4, 18]. IFN-α is used to clean up microscopic tumor cells that may remain in the body following surgery for the disease, and is the only drug that is currently approved for this purpose. In our study, the activation of immune system cells by IFN-α2b treatment appears to have induced the development of episcleritis, which was associated with an idiopathic, immune-mediated disease in this patient.

Radiotherapy is an important treatment for cancer. For a considerable time, it was thought that the main mode of action of radiotherapy was irreversible damage to tumor cell DNA, along with some immunosuppressive effects; however, evidence from recent animal studies suggests that radiotherapy supports local anti-tumor immune activity [5, 19]. There is increasing evidence that radiotherapy leads to alterations in the tumor microenvironment, particularly with respect to the immune infiltrate [7, 19]. Gupta *et al.* [20] found that radiotherapy promotes the activation of tumor-specific effector CD8^+^ T cells via dendritic cell. Our patient’s second episode of episcleritis was closely correlated with the administration of radiotherapy, which appears to have activated the immune system.

## Conclusion

Taken together, these data strongly suggest that the two episodes of episcleritis in this patient with MMHN are due to treatment with IFN-α2b and radiotherapy, which increased the level of CD3^+^ T cells and activated the immune system. However, the molecular mechanism of the effect of IFN-α2b and radiotherapy on episcleritis is still unclear, which is why it is necessary to study further in the future.

## Abbreviations

IFN-α2b: Interferon alfa-2b
MMHN: Mucosal melanoma of the head and neck

## References

[1] Lourenço SV, Fernandes JD, Hsieh R, Coutinho-Camillo CM, Bologna S, Sangueza M, *et al.* Head and neck mucosal melanoma: a review. Am J Dermatopathol. 2014;36:578–87. 10.1097/DAD.0000000000000035 24423929

[2] Ascierto PA, Accorona R, Botti G, Farina D, Fossati P, Gatta G, *et al.* Mucosal melanoma of the head and neck. Crit Rev Oncol Hematol. 2017;112:136–52. 10.1016/j.critrevonc.2017.01.019 28325255

[3] Lahaye MJ, Engelen SM, Nelemans PJ, Beets GL, van de Velde CJ, van Engelshoven JM, *et al.* Imaging for predicting the risk factors--the circumferential resection margin and nodal disease--of local recurrence in rectal cancer: a meta-analysis. Semin Ultrasound CT MR. 2005;26:259–68. 10.1053/j.sult.2005.04.005 16152740

[4] Tarhini AA, Kirkwood JM. Clinical and immunologic basis of interferon therapy in melanoma. Ann N Y Acad Sci. 2009;1182:47–57. 10.1111/j.1749-6632.2009.05073.x PMC3428067 20074274

[5] Sharma A, Bode B, Moch H, Moch H, Okoniewski M, Knuth A, *et al.* Radiotherapy of human sarcoma promotes an intratumoral immune effector signature. Clin Cancer Res. 2013;19:4843–53. 10.1158/1078-0432.CCR-13-0352 23861514

[6] Chatziralli IP, Kanonidou E, Chatzirallis A, Dimitriadis P, Keryttopoulos P. Episcleritis Related to Drug-Induced Lupus Erythematosus following Infliximab Therapy: A Case Report. Case Rep Med. 2011;2011:696285. 10.1155/2011/696285 PMC3087414 21547267

[7] Rastrelli M, Alaibac M, Stramare R, Chiarion Sileni V, Montesco MC, Vecchiato A, *et al.* Melanoma m (zero): diagnosis and therapy. ISRN Dermatol. 2013;2013:616170. 10.1155/2013/616170 PMC3649440 23691346

[8] Rastrelli M, Tropea S, Rossi CR, Alaibac M. Melanoma: epidemiology, risk factors, pathogenesis, diagnosis and classification. In Vivo. 2014;28:1005–11. 25398793

[9] Sera F, Gandini S, Cattaruzza MS, Pasquini P, Picconi O, Boyle P, Melchi CF. Meta-analysis of risk factors for cutaneous melanoma: II. Sun exposure Eur J Cancer. 2015;41:45–60. 10.1016/j.ejca.2004.10.016 15617990

[10] Mendenhall WM, Amdur RJ, Hinerman RW, Werning JW, Villaret DB, Mendenhall NP. Head and neck mucosal melanoma. Am J Clin Oncol. 2005;28:626–30. 10.1097/01.coc.0000170805.14058.d3 16317276

[11] Amit M, Tam S, Abdelmeguid AS, Roberts DB, Raza SM, Su SY, *et al.* Approaches to regional lymph node metastasis in patients with head and neck mucosal melanoma. Cancer. 2018;124:514–20. 10.1002/cncr.31083 29044491

[12] Sun CZ, Li QL, Hu ZD, Jiang YE, Song M, Yang AK. Treatment and prognosis in sinonasal mucosal melanoma: A retrospective analysis of 65 patients from a single cancer center. Head Neck. 2014;36:675–81. 10.1002/hed.23355 23606507

[13] Bartell HL, Bedikian AY, Papadopoulos NE, Dett TK, Ballo MT, Myers JN, *et al.* Biochemotherapy in patients with advanced head and neck mucosal melanoma. Head Neck. 2008;30:1592–8. 10.1002/hed.20910 18798304

[14] Tappeiner C, Walscheid K, Heiligenhaus A. Diagnosis and treatment of episcleritis and scleritis. Ophthalmologe. 2016;113:797–810. 10.1007/s00347-016-0344-3 27550224

[15] Jabs DA, Mudun A, Dunn JP, Marsh MJ. Episcleritis and scleritis: clinical features and treatment results. Am J Ophthalmol. 2000;130:469–76. 10.1016/s0002-9394(00)00710-8 11024419

[16] De Andrea M, Ravera R, Gioia D, Gariglio M, Landolfo S. The interferon system: an overview. Eur J Paediatr Neurol. 2002;6 Suppl A:A41–6. discussion A55–8 10.1053/ejpn.2002.0573 12365360

[17] Colonna M, Trinchieri G, Liu YJ. Plasmacytoid dendritic cells in immunity. Nat Immunol. 2004;5:1219–26. 10.1038/ni1141 15549123

[18] Anonymous. IFN-alpha found to act on immune-system cells rather than melanoma cells. Future Oncol. 2007;3:505. 10.2217/14796694.3.5.505 17927515

[19] Yoshimoto Y, Suzuki Y, Mimura K, Ando K, Oike T, Sato H, *et al.* Radiotherapy-induced anti-tumor immunity contributes to the therapeutic efficacy of irradiation and can be augmented by CTLA-4 blockade in a mouse model. PLoS One. 2014;9:e92572. 10.1371/journal.pone.0092572 PMC3970971 24686897

[20] Gupta A, Probst HC, Vuong V, Landshammer A, Muth S, Yagita H, *et al.* Radiotherapy promotes tumor-specific effector CD8+ T cells via dendritic cell activation. J Immunol. 2012;189:558–66. 10.4049/jimmunol.1200563 22685313

